# Solving the Puzzle: Connecting a Heterologous *Agrobacterium tumefaciens* T6SS Effector to a *Pseudomonas aeruginosa* Spike Complex

**DOI:** 10.3389/fcimb.2020.00291

**Published:** 2020-06-23

**Authors:** Sarah Wettstadt, Erh-Min Lai, Alain Filloux

**Affiliations:** ^1^MRC Centre for Molecular Bacteriology and Infection, Department of Life Sciences, Imperial College London, London, United Kingdom; ^2^Institute of Plant and Microbial Biology, Academia Sinica, Taipei, Taiwan

**Keywords:** type VI secretion system, bacterial toxin, VgrG, PAAR, Tde1

## Abstract

The type VI secretion system (T6SS) is a contractile injection apparatus that translocates a spike loaded with various effectors directly into eukaryotic and prokaryotic target cells. Such T6SS spike consists of a needle-shaped trimer of VgrG proteins topped by a conical and sharp PAAR protein that facilitates puncturing of the target membrane. T6SS-delivered effector proteins can be either fused to one of the two spike proteins or interact with either in a highly specific manner. In *Agrobacterium tumefaciens* the T6SS effector Tde1 is targeted to its cognate VgrG1 protein. Here, we attempted to use a VgrG shuttle to deliver a heterologous T6SS effector by directing Tde1 onto a T6SS spike in *Pseudomonas aeruginosa*. For this, we designed chimeras between VgrG1 from *A. tumefaciens* and VgrG1a from *P. aeruginosa* and showed that modification of the spike protein hampered T6SS functionality in the presence of the Tde1 effector complex. We provide evidence suggesting that Tde1 specifically binds to the VgrG spike in the heterologous environment and propose that there are additional requirements to allow proper effector delivery and translocation. Our work sheds light on complex aspects of the molecular mechanisms of T6SS delivery and highlights some limitations on how effectors can be translocated using this nanomachine.

## Introduction

The T6SS is a versatile secretion system, injecting effector proteins into target cells which equips bacteria with the ability to establish a niche in any given polymicrobial environment or modulate host cell responses. The T6SS is anchored to the bacterial cytoplasmic membrane *via* a so-called membrane complex (Durand et al., 2012, 2015) which is connected to a cytosolic membrane bound baseplate (Brunet et al., 2015; Planamente et al., 2016). The cytosolic tubular sheath attaches to the baseplate at the inner membrane and encompasses a tube composed of Hcp hexamers that is propelled out of the cell upon sheath contraction (Pukatzki et al., 2006; Leiman et al., 2009; Brunet et al., 2014). On top of the Hcp tube and residing within the baseplate complex sits the so-called T6SS spike consisting of a needle-shaped trimer of VgrG proteins and a conically-shaped PAAR protein (Shneider et al., 2013). The VgrG protein consists of a gp5- and a gp27-like domain that, when assembled to a trimer, form a rigid structure due to the intertwining of the C-terminal hydrophobic β-sheets (Kanamaru et al., 2002). Each last β-sheet binds to the hydrophobic surface of a cognate PAAR protein (Shneider et al., 2013). The VgrG-PAAR spike complex has two main functions: it facilitates puncturing of target membranes while it is also directly involved in carrying T6SS effectors into the target cell (Shneider et al., 2013).

T6SS effectors are classified into two groups: specialized effectors and cargo effectors (Durand et al., 2014). A specialized, or evolved, effector contains an N-terminal domain that is a structural component, like VgrG, PAAR, or Hcp, essential for T6SS assembly. The C-terminal domain, however, is an extension with an effector domain and is not required for delivery of the VgrG-PAAR spike complex (Ma et al., 2009; Wood et al., 2019b). In a different scenario, cargo effectors interact non-covalently with structural components, like Hcp, VgrG, or PAAR, and once the T6SS propels out the spike, the cargo effector is delivered in a “piggy-back ride” (Hachani et al., 2014). This so-called “á* la carte*” effector delivery concept describes that a VgrG recruits and delivers one specific effector (Hachani et al., 2014) and that this specific interaction is mediated by the C-terminal residues or even C-terminal domains of VgrG proteins. This interaction is the prerequisite for effector delivery and was shown for Tde1 binding VgrG1 in *Agrobacterium tumefaciens*, Tle1 binding VgrG1 in enteroaggregative *Escherichia coli* and PldA and PldB binding VgrG4b and VgrG5, respectively, in *P. aeruginosa*, amongst others (Flaugnatti et al., 2015; Unterweger et al., 2015; Bondage et al., 2016; Wettstadt et al., 2019).

Furthermore, some T6SS effectors were identified that require additional proteins for their delivery, which can be of the DUF1795, DUF2169, or DUF4123 family (Diniz and Coulthurst, 2015; Liang et al., 2015; Unterweger et al., 2015; Bondage et al., 2016; Quentin et al., 2018). *DUF4123* genes, also coined *tap* (Unterweger et al., 2015), or *tec* (Liang et al., 2015), can be found in the vicinity of a range of T6SS effector-encoding genes, together with a gene encoding a VgrG or PAAR, mediating delivery of the effector. Tap components are proven to be essential for the delivery of a range of effectors, like Tde1 from *A. tumefaciens*, TseL from *Vibrio cholerae*, or TseF from *P. aeruginosa* (Liang et al., 2015; Unterweger et al., 2015; Bondage et al., 2016). The current model suggests that Tap binds and stabilizes its cognate effector. Tap then facilitates binding of the effector to the C-terminus of the cognate VgrG or PAAR protein and subsequently dissociates from the tip. After dissociation of Tap, the effector remains bound to the VgrG or PAAR protein in a yet unknown mechanism but upon sheath contraction and by pushing the spike complex in the cell envelope, the effector is then transported (Bondage et al., 2016; Burkinshaw et al., 2018).

To broaden our knowledge on the molecular mechanisms of effector delivery, our aim was to achieve heterologous effector delivery. We used the nuclease effector Tde1 from *A. tumefaciens* and attempted to connect it to the VgrG1a spike in *P. aeruginosa*. For this, we constructed VgrG1a chimeras containing the C-terminal Tde1-binding extension from the *A. tumefaciens* VgrG1. We could show that these chimeras bind the cognate Tde1, however effector delivery could not be attained. This highlights the specificity of the T6SS spike for its effectors and outlines limitations for T6SS-mediated effector delivery.

## Materials and Methods

### Bacterial Strains and Growth Conditions

Bacterial strains used in this study are described in Table S1. *P. aeruginosa* strains were grown in tryptone soy broth (TSB) or LB supplemented with antibiotics where appropriate (spectinomycin 2,000 μg mL^−1^) at 37°C with agitation. *Escherichia coli* strains were grown in LB broth supplemented with antibiotics where appropriate (streptomycin 50 μg mL^−1^, kanamycin 50 μg mL^−1^). *A. tumefaciens* was grown at 28°C in minimal medium as described before (Lin et al., 2013).

### DNA Manipulation

DNA purification was performed using the PureLink Genomic DNA minikit (Life Technologies) while plasmid DNA isolation using the QIAprep spin miniprep kit (Qiagen). Restriction endonucleases were used according to the manufacturer's specifications (New England Biolabs or Roche) and all used oligonucleotides are listed in Table S2 and were purchased from Sigma. KOD Hot Start DNA Polymerase (Novagen) was used to amplify genes or DNA fragments used for the construction of mutator plasmids and deletion mutants as described by the manufacturer with the inclusion of 0.5 M betaine (Sigma). Colony PCR was performed with Taq polymerase (New England Biolabs) and DNA sequencing was performed by GATC Biotech.

### Construction of *P. aeruginosa* Mutants

*P. aeruginosa* deletion mutants were constructed as described previously (Vasseur et al., 2005; Ventre et al., 2006) using the suicide plasmid pKNG101 (Herrero et al., 1990; Kaniga et al., 1991). Briefly, to create PAO1Δ*tse6tsi6*, 500-bp DNA fragments of the 5′ (up) and 3′ (down) ends of the *tse6-tsi6* gene pair were obtained by PCR using PAO1 chromosomal DNA as a template with the oligonucleotides Δ*tse6tsi6*_upF and Δ*tse6tsi6*_upR as well as with Δ*tse6tsi6_*dnF and Δ*tse6tsi6_*dnR (Table S3). A third PCR step using Δ*tse6tsi6*_upF and Δ*tse6tsi6_*dnR resulted in a DNA fragment with a clean deletion of the *tse6-tsi6* gene pair. To create the chimeric *vgrG1a* genes, splicing by overlap extension PCRs was performed initiated by three single PCR fragments. Gene fragments containing ~500 bp upstream and downstream of the splice junction were amplified using the overlapping primers *construct*_upR and *construct*_dnF as well as the upstream *vgrG1a*_F and downstream *vgrG1a*_R primers from the *P. aeruginosa* genome. A third gene fragment containing the fusion fragment of interest was obtained by using primers *construct*_upF and *construct*_dnR that are overlapping with *construct*_upR and *construct*_dnF, respectively. Subsequently, two overlap extension PCR steps were undertaken, employing an equimolar ratio of the upstream and downstream fragments as the DNA template. The gene fragments were cloned into pCR-BluntII-TOPO (Invitrogen), their sequences confirmed and sub-cloned into the pKNG101 suicide vector (Table S2). The pKNG-derivatives were maintained in *E. coli* strain CC118λpir and mobilized into *P. aeruginosa* PAK using *E. coli* 1,047 carrying the conjugative plasmid pRK2013 (Figurski and Helinski, 1979). Clones, in which double recombination events occurred, resulting in the deletion of the gene of interest *(GOI)* or fusion to *GOI*, were isolated using counterselection on sucrose plates as previously described (Vasseur et al., 2005). Gene deletions were verified by PCR using external primers and gene fusions confirmed by sequencing.

### Secretion Assay

Secretion assays were performed as previously described (Hachani et al., 2011). Bacterial suspension was diluted from overnight cultures in TSB to OD_600_ of 0.1 and grown at 37°C to an OD_600_ of 5. A bacterial culture sample adjusted to OD_600_ of 1 was harvested by centrifugation and served as the whole cell sample. Thirteen milliliters of culture was centrifuged at 4,000 g for 20 min at 4°C to separate the bacterial cells and the culture supernatant. Then, 10 mL of the supernatant was transferred into falcon tubes and centrifuged again; 7 mL of the uppermost supernatant was transferred into new tubes and centrifuged. Two hundred microliters trichloroacetic acid were added to 1.8 mL supernatant fraction to precipitate proteins overnight at 4°C. The protein precipitate was obtained by centrifugation at 16,000 g for 30 min at 4°C, washed with cold 90% (v/v) acetone and centrifuged one more time. After removing the supernatant, the washed pellet was air-dried for 30 min and resuspended in 1x Laemmli buffer to an OD_600_ equivalent of 10.

### Western Blot Analysis and SDS-PAGE

For SDS-PAGE analysis, cell extracts were loaded/migrated onto SDS polyacrylamide gels, and proteins transferred to a nitrocellulose membrane at 3 mA/cm^2^. Following transfer, membranes were incubated overnight in blocking buffer (5% milk powder, 0.1% Tween 20 in Tris-buffered saline, pH 8.0). Polyclonal antibodies against VgrG1abc were used at a dilution of 1:1,000 (Hachani et al., 2011), Hcp1 at 1:1,000 (Hachani et al., 2011), Tde1 at 1:1,000 (Bondage et al., 2016), VgrG1^*Atu*^ at 1:1,000 (Bondage et al., 2016), and Tse3 at 1:500 (Hachani et al., 2011). Monoclonal antibodies against the β subunit of RNA polymerase (RpoB, NeoClone) were used at 1:5,000. Secondary antibodies conjugated to horseradish peroxidase were used at a dilution of 1:5,000. Western blots were developed using Super-Signal West Pico Chemiluminescent Substrate (Pierce) and visualized on a LAS3000 Fuji Imager.

### Relative Protein Quantification

The intensities of the bands corresponding to the VgrG1a-chimeras were analyzed using the intensity measurement tool in the software ImageJ. Figures from three independent experiments were analyzed. The band corresponding to VgrG1a in wild-type (WT) cells was set as a standard for VgrG1a content in the cell. All measured intensities for other bands were divided by the intensity for VgrG1a under WT conditions. RpoB was used as protein loading control.

### Interbacterial Competition Assays

Interbacterial competition assays were conducted on solid media due to the contact-dependent killing of the T6SS. Prey *P. aeruginosa* strains contained the Mini-CTX-*lacZ* integrated at the *att* site, consequently resulting in blue colonies on X-gal-containing plates. Overnight cultures in TSB were collected by centrifugation at 8,000 g for 3 min before washing twice in 1 mL sterile PBS and normalized to OD_600_ of 1.0. The OD_600_ was measured again for confirmation and 100 μL of attacker and 20 μL prey strains were mixed. This mixture was centrifuged at 8,000 g for 3 min and 20 μL supernatant was removed to give a competition mixture ratio of 5:1 of attacker and prey strains. Twenty microliters of each competition mix was spotted in duplicates onto LB-agar, the spots dried and the Petri dish lids were secured using parafilm M (Bemis). Competition plates were inverted and incubated at 37°C for 5 h for H1-T6SS-inducive killing.

The input competitions were serially diluted to 10^−7^, plated on selective media for both attacker and prey (LB agar with 100 μg mL^−1^ X-gal for blue/white *P. aeruginosa* prey/attacker differentiation) and grown overnight at 37°C to confirm the input ratios. Competition spots were collected using 10 μL inoculation loops (VWR) and resuspended in 1 mL PBS. The competition output mixture was serially diluted to 10^−7^, plated on selective media and grown overnight at 37°C similarly to the input. Both attacker and prey colony forming units were enumerated on both input and output dilution plates. All competition assays were repeated three times unless otherwise stated and the mean colony forming units (cfu) of survived prey strains obtained from all experiments with the standard deviation was plotted.

### Bacterial-Two-Hybrid Assay

Genes expressing *tde1* or *tap1-tde1* were cloned into pUT18C and pKT25 and sequence confirmed. Variations of both vectors were introduced into *E. coli* DHM1 via heat shock and selected on LB plates containing both Kanamycin and Ampicillin. Resulting colonies were picked and grown in LB containing both antibiotics and overnight cultures were spotted onto LB containing Kanamycin (100 μg μL^−1^), Ampicillin (100 μg μL^−1^), IPTG (1 μM), and X-gal (100 μg μL^−1^) and grown for 48 h at 30°C.

## Results

### Heterologous Secretion of Tde1 From *A. tumefaciens*

In this study, we meant to investigate whether a heterologous T6SS cargo effector could be delivered by a T6SS solely by manipulating the VgrG tip. We chose Tde1 from *A. tumefaciens* as the heterologous effector, firstly because the Tde1 orthologs are only found in a-proteobacteria and secondly because, its basic delivery system has previously been studied in great details (Ma et al., 2014; Bondage et al., 2016; Wu et al., 2020). The current model states that VgrG1 from *A. tumefaciens* (VgrG1^*A*^) is assembled to a functional trimer and capped by the cognate PAAR protein, with one PAAR protein binding the three last VgrG1^*A*^ β-strands. Concomitantly, Tap1 interacts with and stabilizes Tde1 within the cell and the Tap1-Tde1 complex is recruited to the C-terminal amino acids of VgrG1^*A*^. Since Tap1 was not detected in the supernatant fraction, it is believed to be released prior to secretion, while Tde1 remains bound to the VgrG1^*A*^ spike, which is propelled out of the cell (Bondage et al., 2016).

**Figure 1:**
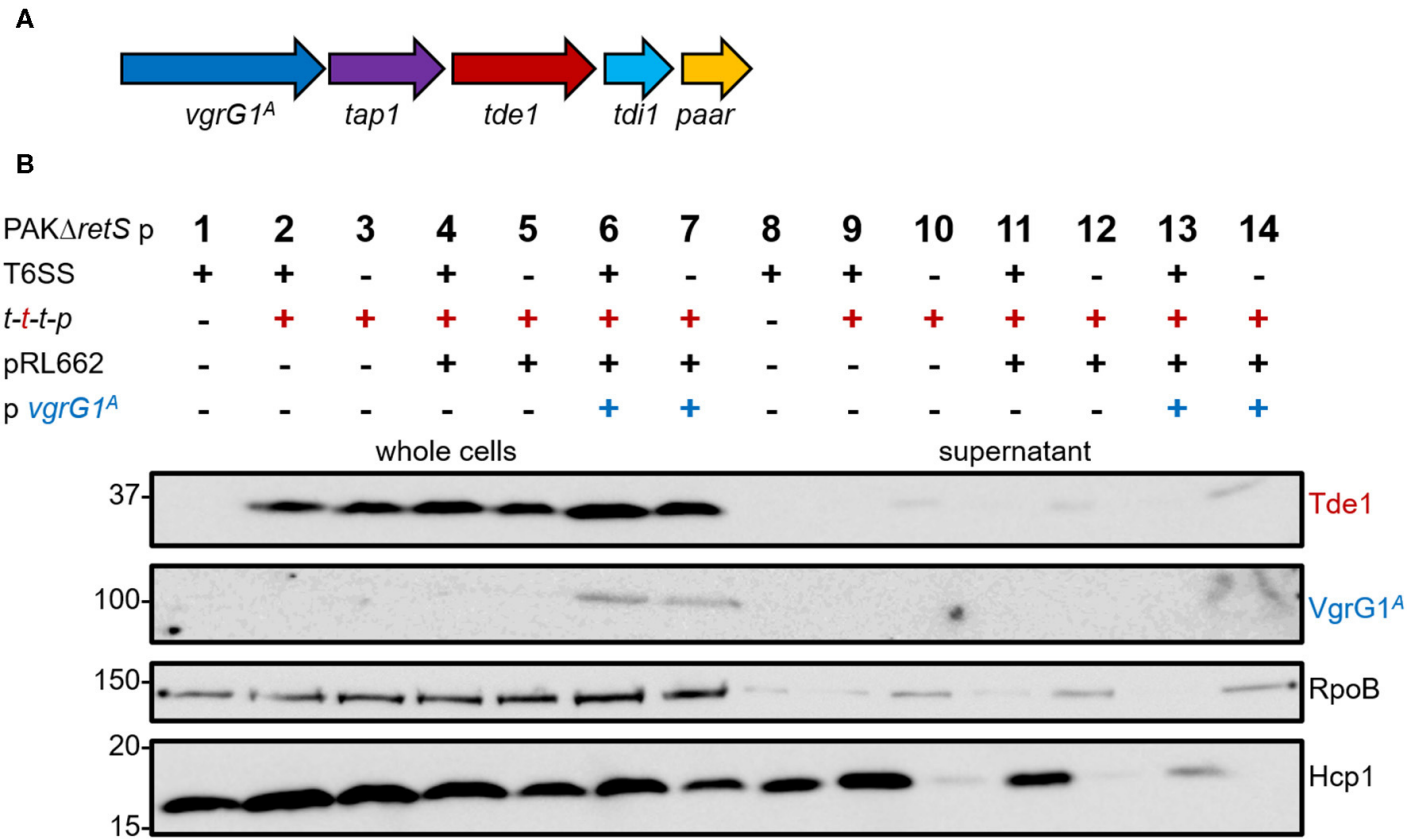
*P. aeruginosa* cannot perform heterologous secretion of *A. tumefaciens* VgrG1^*A*^ and Tde1. **(A)** The set of genes *tap1-tde1-tdi1-paar* (purple-red-cyan-orange) were expressed from pTrc200, while *vgrG1*^*A*^ (blue) was expressed from pRL662 in *P. aeruginosa* (Bondage et al., 2016). **(B)** Representative figure of a western blot from a secretion assay of PAKΔ*retS* pTrc200 (p) with an active (+) or inactive (–) H1-T6SS expressing (+) *tap1-tde1-tdi1-paar* (*t-t-t-p*) or containing pRL662 (+) with *vgrG1*^*A*^ (+). Antibodies used (from top to bottom) are against Tde1, VgrG1^A^, RpoB, and Hcp1 as indicated on the right.

We aimed at directing the heterologous effector Tde1 toward VgrG1a from the H1-T6SS in *P. aeruginosa* (VgrG1a^*P*^). To assess whether *P. aeruginosa* is naturally able to deliver Tde1 using its H1-T6SS, we introduced the plasmid pTrc200 containing the *A. tumefaciens tap1-tde1-tdi1-paar* (t-t-t-p) (Figure 1A) into *P. aeruginosa* PAKΔ*retS*, which has an active H1-T6SS, and performed secretion assays (Figure 1B). For all following experiments, we used a plasmid expressing a catalytic mutant of Tde1, as *P. aeruginosa* growth was inhibited when cells expressed WT Tde1 likely due to Tde1 toxicity (Ma et al., 2014). Tde1 (Figure 1B, top panel) is expressed in consistent amounts (lanes 2 and 3), and no secretion is observed in a T6SS-positive background (lane 9). Intriguingly, a weak Tde1 band is detectable in the supernatant fraction of T6SS-inactive strains (lane 10) but this also correlates with elevated detection of RpoB in this fraction (third panel) and suggests partial cell lysis. We then attempted to connect Tde1 onto the *P. aeruginosa* T6SS by co-expressing the cognate *A. tumefaciens vgrG1*^*A*^ (Figure 1B, second panel). In this case, we observed that the bands corresponding to Tde1 increased in intensity (lanes 6 and 7), which suggests that a specific interaction between Tde1 and VgrG1^*A*^ may occur in *P. aeruginosa* resulting in Tde1 stabilization. However, neither VgrG1^*A*^ nor Tde1 could be detected in the supernatant fractions (lanes 13) suggesting no efficient Tde1 secretion even in the presence of its cognate VgrG. Again, some traces of Tde1 are found in the supernatant of the T6SS-inactive background (lane 14) but it correlates with elevated RpoB levels. Interestingly, a decreased amount of Hcp1 in the supernatant fraction (lane 13) could clearly be seen which suggests that the H1-T6SS function is altered in the presence of VgrG1^*A*^, possibly because the heterologous VgrG is able to partially connect to the *P. aeruginosa* T6SS but is then not further engaged in the secretion process.

### Design of VgrG Chimeras to Connect Tde1 to the H1-T6SS

**Figure 2:**
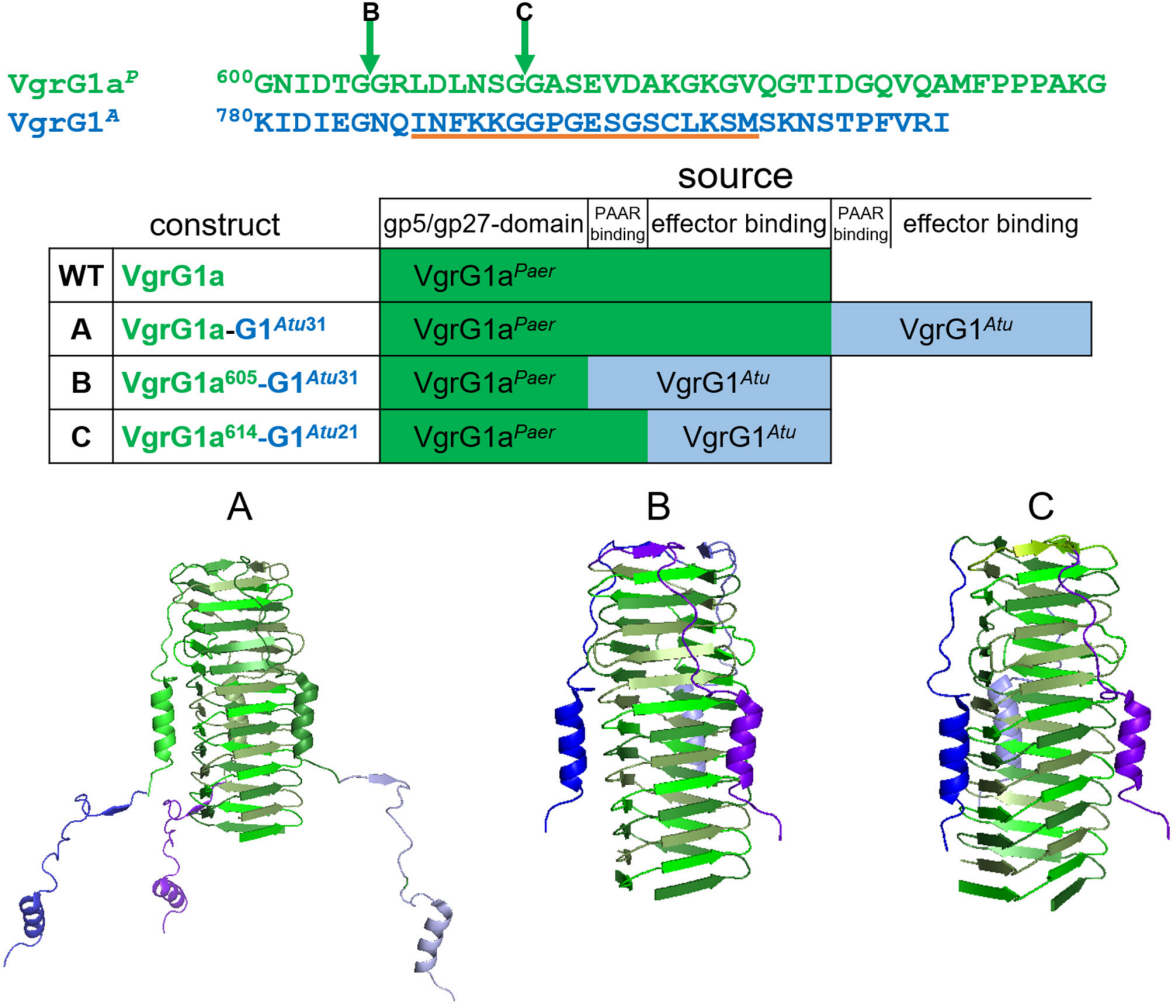
Construction of VgrG chimeras. The top panel represents the amino acid sequences of the C-terminal extensions of VgrG1a^*P*^ (green) and VgrG1^*A*^ (blue). The arrows correspond to the swapping points after amino acid (aa) 605 in chimera B and aa 614 in chimera C. The orange line corresponds to the epitope, against which the VgrG1 antibody was raised. In the lower panel structural models (pdb:4mtk) of the needle parts of the three designed chimeras are shown. In construct A, the STOP codon of *vgrG1a*^*P*^ (green) was replaced by the gene portion corresponding to the last 31 aa of VgrG1^*A*^ (blue). Constructs B,C were designed by substituting the gene portions corresponding to the last 35 aa and 29 aa of *vgrG1a*^*P*^ with the gene portions corresponding to the last 31 aa and 21 aa, respectively, of VgrG1^*A*^.

As an alternative to using the entire *A. tumefaciens* VgrG1^*A*^, we decided to design chimeras to directly connect Tde1 to the *P. aeruginosa* VgrG1a. It was established that the 31 C-terminal amino acids of VgrG1^*A*^ is required for binding the Tap1-Tde1-complex and a prerequisite for Tde1 delivery in *A. tumefaciens* (Bondage et al., 2016). We designed three chimeras, which we named A, B, and C, between VgrG1a^*P*^ from *P. aeruginosa* (Figure 2, green) and VgrG1^*A*^ from *A. tumefaciens* (Figure 2, blue) to connect Tde1 to the *P. aeruginosa* H1-T6SS as shown in Figure 2, while any of the chimera would replace the WT *vgrG1a* gene on the chromosome. Construct A (VgrG1a^*P*^-G1^A31^) contains the full length VgrG1a^*P*^ extended by 31 C-terminal amino acids from VgrG1^*A*^, thus including amino acids likely responsible for binding the cognate *A. tumefaciens* PAAR and Tde1 (Bondage et al., 2016). Construct B (VgrG1a^P605^-G1^A31^) covers the first 605 amino acids from VgrG1a^*P*^ and 31 C-terminal amino acids from VgrG1^*A*^, while construct C (VgrG1a^P614^-G1^A21^) comprises of the first 614 amino acids from VgrG1a^*P*^ and 21 C-terminal amino acids from VgrG1^*A*^.

**Figure 3:**
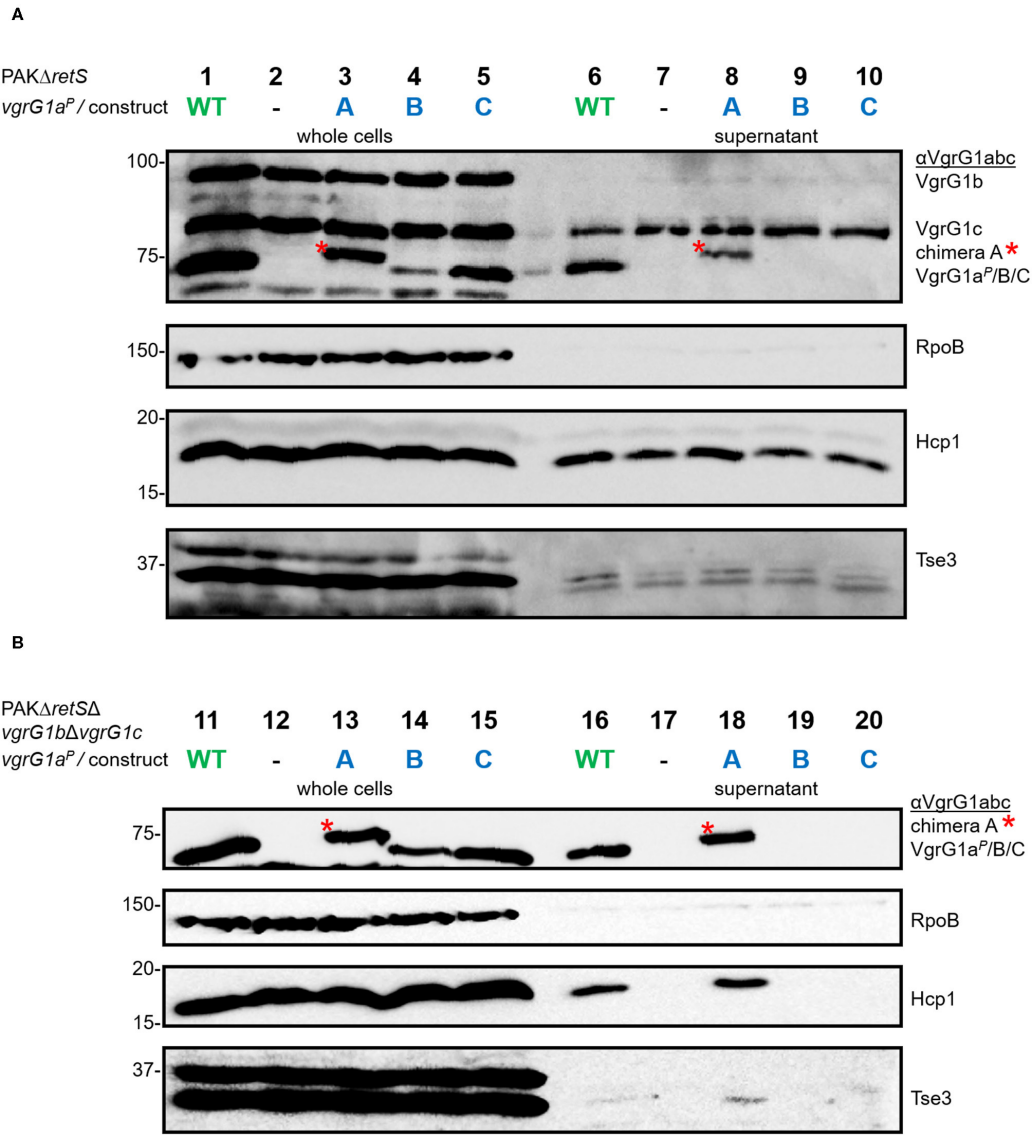
Modifying the C-terminus of VgrG1a inhibits T6SS functionality. Representative figures of western blots from three independent secretion assays of **(A)** PAKΔ*retS* and **(B)** PAKΔ*retS*Δ*vgrG1b*Δ*vgrG1c* expressing Wild type VgrG1a^*P*^ (WT), no VgrG1a^*P*^ (–) or the chimeras A, B, C between VgrG1a^*P*^ and VgrG1^*A*^. Antibodies used (from top to bottom) are against VgrG1abc, RpoB, Hcp1, and the Hcp1-dependent effector Tse3 as indicated on the right.

First, we investigated whether modification of the VgrG1a spike protein impacts its ability to form a functional T6SS spike and therefore its secretion. We integrated the chimeras into PAKΔ*retS* background and performed secretion assays (Figure 3A) and detected all three chimeras with an antibody against VgrG1abc (top panel, lanes 3–5). While chimera B seemed to be less stable within the whole cell fraction, we only detected chimera A in the supernatant fraction (lane 8) suggesting it to be secreted. We then asked whether the chimeras would also be stable in absence of the other two VgrG proteins VgrG1b and VgrG1c and whether their absence would impact secretion of any of the chimeras. Using PAKΔ*retS*Δ*vgrG1b*Δ*vgrG1c* cells (Figure 3B), thus lacking the two other VgrGs related to the H1-T6SS, we confirmed data from previous studies showing that VgrG1a on its own is able to form a functional T6SS spike promoting efficient Hcp1 secretion, as well as secretion of the Hcp1-dependent effector Tse3 (Figure 3B, lane 16) (Hachani et al., 2014). When testing any of the VgrG1a chimeras, only VgrG1a-construct A expressing full length VgrG1a^*P*^ fused to G1^A31^ is able to execute this function as seen by identifying Hcp1, VgrG1a^*P*^-G1^A31^, and Tse3 in the supernatant (Figure 3B, lane 18, red asterisk). None of the other two chimeras seems to be able to form a functional T6SS spike.

### Connecting Tde1 to the VgrG1a Spike

**Figure 4:**
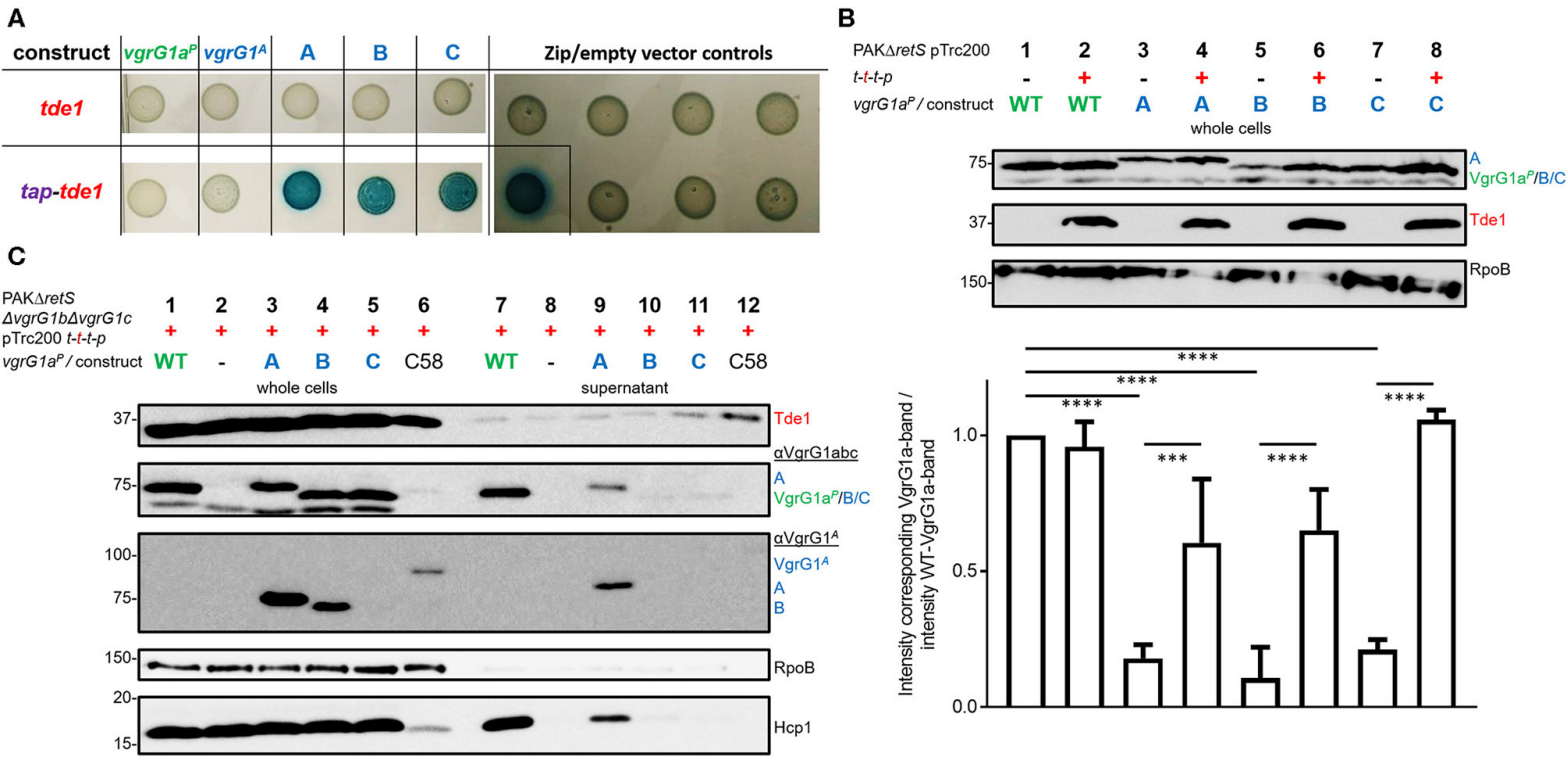
All chimeras interact with Tde1 but are unable to facilitate effector secretion. **(A)** T25 and T18-domains were fused to the C-termini of either VgrG or a catalytic mutant of Tde1 (red) in presence of the cognate Tap1 (purple). Representative bacterial-two-hybrid plates tested for interactions between VgrG1a^*P*^-G1^*A*^-chimeras and Tde1 on its own (top panel) or in presence of the Tap1 protein (bottom panel) (Bondage et al., 2016). All interactions were tested with all gene sequences cloned into both pKT25 and pUT18C represented as two columns for each tested interaction. Empty vector controls as well as the leucine-*zipper* pUT18C-*Zip* and pKT25-*Zip* vectors are shown in the right panel. **(B)** Top panel: Representative western blot of whole cell lysates of PAKΔ*retS* containing pTrc200 and expressing chimera A, B, C between VgrG1a^*P*^ and VgrG1^*A*^ in absence (–) and presence (+) of *tap1-tde1-tdi1-paar* (*t-t-t-p*). As a control for VgrG1a^*P*^ stability, the parental strain was included and grown in absence and presence of *t-t-t-p* (lanes 1 and 2). Antibodies were used against VgrG1abc, Tde1, and RpoB as indicated on the right. Bottom panel: Intensity of the corresponding VgrG1a^*P*^ bands from three independent experiments was quantified using ImageJ (https://imagej.nih.gov/ij/index.html) and divided by the intensity of the band belonging to WT VgrG1a^*P*^ in absence of Tde1 (lane 1) as a standard for VgrG1a stability. One-Way ANOVA with Tukey's multiple comparison test was conducted between datasets as indicated with ****p < 0.0001. **(C)** Representative figures of western blots from three independent secretion assays of PAKΔ*retS*Δ*vgrG1b*Δ*vgrG1c* pTrc200 expressing chimeras A, B, C between VgrG1a^*P*^ and VgrG1^*A*^ in presence (+) of *tap1-tde1-tdi1-paar (t-t-t-p)*. As a control for Tde1 secretion the supernatant fraction of *A. tumefaciens* C58 was included (lanes 6 and 12). Antibodies (from top to bottom) against Tde1, VgrG1abc, VgrG1^*A*^, RpoB, and Hcp1 were used as indicated. ***p < 0.001.

We then assessed whether Tde1 could bind to the C-terminus of VgrG1^*A*^, when the latter is fused to a heterologous VgrG vehicle as is the case in our three chimeric constructs. We used a bacterial-two-hybrid (BTH) assay, in which the three chimeras as well as a catalytic Tde1 mutant carry the T18- or T25-domains (T18/25) at their C-termini. When testing interactions between Tde1 and any of the VgrG-constructs, no blue colonies appeared indicating that the two proteins do not interact (Figure 4A, top panel). Since Tde1 only binds VgrG1^*A*^ in presence of Tap1 in *A. tumefaciens* (Bondage et al., 2016), we then cloned the *tap1* gene upstream of *tde1* and re-tested the interactions (bottom panel). When Tap1 is present, dark blue spots could be readily observed suggesting strong interactions between Tap1-Tde1 and any of the VgrG1a^*P*^-G1^*A*^-chimeras. The fact that construct C efficiently associates with Tap1-Tde1 suggests that as little as the 21 C-terminal amino acids of VgrG1^*A*^ are sufficient to mediate this interaction. Intriguingly, Tap1-Tde1 binds only very weakly to VgrG1^*A*^, which might be due to the lack of the cognate PAAR (Bondage et al., 2016).

This result suggests that Tap1-Tde1 could recognize the C-terminus of VgrG1^*A*^ when it is plugged onto a heterologous VgrG core. We then investigated whether this interaction would also occur in *P. aeruginosa* cells, which would be the prerequisite for Tde1 secretion. For this, PAKΔ*retS* cells expressing WT VgrG1a^*P*^ or any of the chimeras (A/B/C) were grown for 5 h at 37°C in the presence (+) or absence (–) of a plasmid carrying the *tap1-tde1-tdi1-paar (t-t-t-p)* genes. Western blot analysis was performed on whole cell lysates probed for Tde1 and VgrG1abc (Figure 4B) as well as RpoB as a loading control. Images from three independent experiments were analyzed using ImageJ and the intensity of the VgrG1a^*P*^ band in absence of Tde1 was quantified (bottom plot) and served as a standard for VgrG1a^*P*^ level. The intensity of any bands corresponding to VgrG1a^*P*^/constructABC was quantified and divided by the standard intensity for VgrG1a^*P*^ in Tde1 absence (Figure 4B, lane 1). According to this calculation, the closer a ratio is to 1, more of this protein is present in the cell. No difference in intensity between the VgrG1a^*P*^ bands in absence (Figure 4B, lane 1) or presence (Figure 4B, lane 2) of Tap1-Tde1-Tdi1-PAAR was observed. However, the intensity of the bands representing the VgrG1a^*P*^-G1^*A*^ chimeras shows variability when in presence or absence of Tap1-Tde1-Tdi1-PAAR. Indeed, a drastic decrease in abundance is observed in absence of Tap1-Tde1-Tdi1-PAAR (Figure 4B, lanes 3, 5, and 7) while co-expression of Tap1-Tde1-Tdi1-PAAR led to a significant increase in intensity of the corresponding bands (Figure 4B, lanes 4, 6, and 8). This suggests a lack of stability of the chimeric VgrG in absence of the Tap1-Tde1-Tdi1-PAAR complex. Since PAAR proteins were shown to bind VgrG spike proteins (Shneider et al., 2013), one could suggest this to be the case here. However, chimera C does not contain the interaction motif for PAAR but its stability is the most increased amongst the chimeras (Figure 4B, lane 8). Hence, interactions between the chimeras and the PAAR protein can be ruled out. Furthermore, no immunity protein was ever shown to interact directly with a VgrG protein, so it is unlikely that the immunity Tdi1 would interact with any chimera. Since Tde1 was shown to interact with VgrG1^*A*^ in presence of Tap1 *in vivo* (Bondage et al., 2016), we propose that here a Tap1-Tde1 complex could bind to any of the tested chimeras.

### Connecting Tde1 to the VgrG1a Spike Is Not Sufficient for T6SS-Mediated Secretion

Having established that chimera A is proficiently secreted and that the heterologous effector Tde1 seems to bind to it for stability, we then aimed at testing whether chimera A could be a secretion vehicle to deliver Tde1 from *P. aeruginosa*. In Figure 3 we showed that stability of the chimeras is independent of the presence of the other two VgrG proteins and western blot detection of the chimeras is facilitated in the absence of VgrG1b and VgrG1c due to cross-recognition by the antibody. Furthermore, Hcp1 secretion in the PAKΔ*retS*Δ*vgrG1b*Δ*vgrG1c* background represents an adequate readout for T6SS functionality (Figure 3B), and we chose to test Tde1 secretion from *P. aeruginosa* in the absence of VgrG1b and VgrG1c. Hence, we performed standard secretion assays of *P. aeruginosa* strains that additionally expressed *tap1-tde1-tdi1-paar* genes (Figure 4C). Western blot assays using antibodies against both VgrG1a^*P*^ and an amino acid stretch from the C-terminus of VgrG1^*A*^ (Figure 2, orange line) (Bondage et al., 2016) revealed that VgrG1a^*P*^ and all VgrG1a^*P*^-VgrG1^*A*^-chimeras are produced in significant amounts (Figure 4C, second and third panels, lanes 1, 3–5). We did not detect construct C (lane 5) with the antibody against the C-terminal amino acids of VgrG1^*A*^, which is surprising as this antibody was raised against an amino acid stretch that includes most of the 21 amino acids, hence one might have expected the antibody would recognize our chimera. However, since the antibody against VgrG1abc from *P. aeruginosa* did detect this chimera, we could assume production of the protein. Yet, in the supernatant fractions, we could only detect VgrG1a^*P*^ (lane 7) and construct A (lane 9). We also monitored Hcp1 as a readout for T6SS functionality (bottom panel) but detected this protein only in the supernatant fractions of strains expressing and secreting VgrG1a^*P*^ or construct A. Neither chimera B or C, nor Hcp1, were detected in the supernatant fractions when using strains expressed chimera B or C (lanes 10 and 11). This confirms that only construct A is able to form a functional T6SS tip.

Even though we observed faint bands for Tde1 in the supernatant fractions of all tested strains (Figure 4C, top panel) we suggest it is unlikely resulting from T6SS-dependent secretion since there is no particular increase in the intensity of the Tde1 band in presence of the functional secreted chimera A (lane 9) as compared to other non-secreted chimeras (lanes 10 and 11). As a control for Tde1 secretion, *A. tumefaciens* was grown under T6SS-inducing conditions (Lin et al., 2013) displaying an intense band for Tde1 in the supernatant (lane 12).

### Tde1 Presence Interferes With T6SS Functionality and Effector Delivery

**Figure 5:**
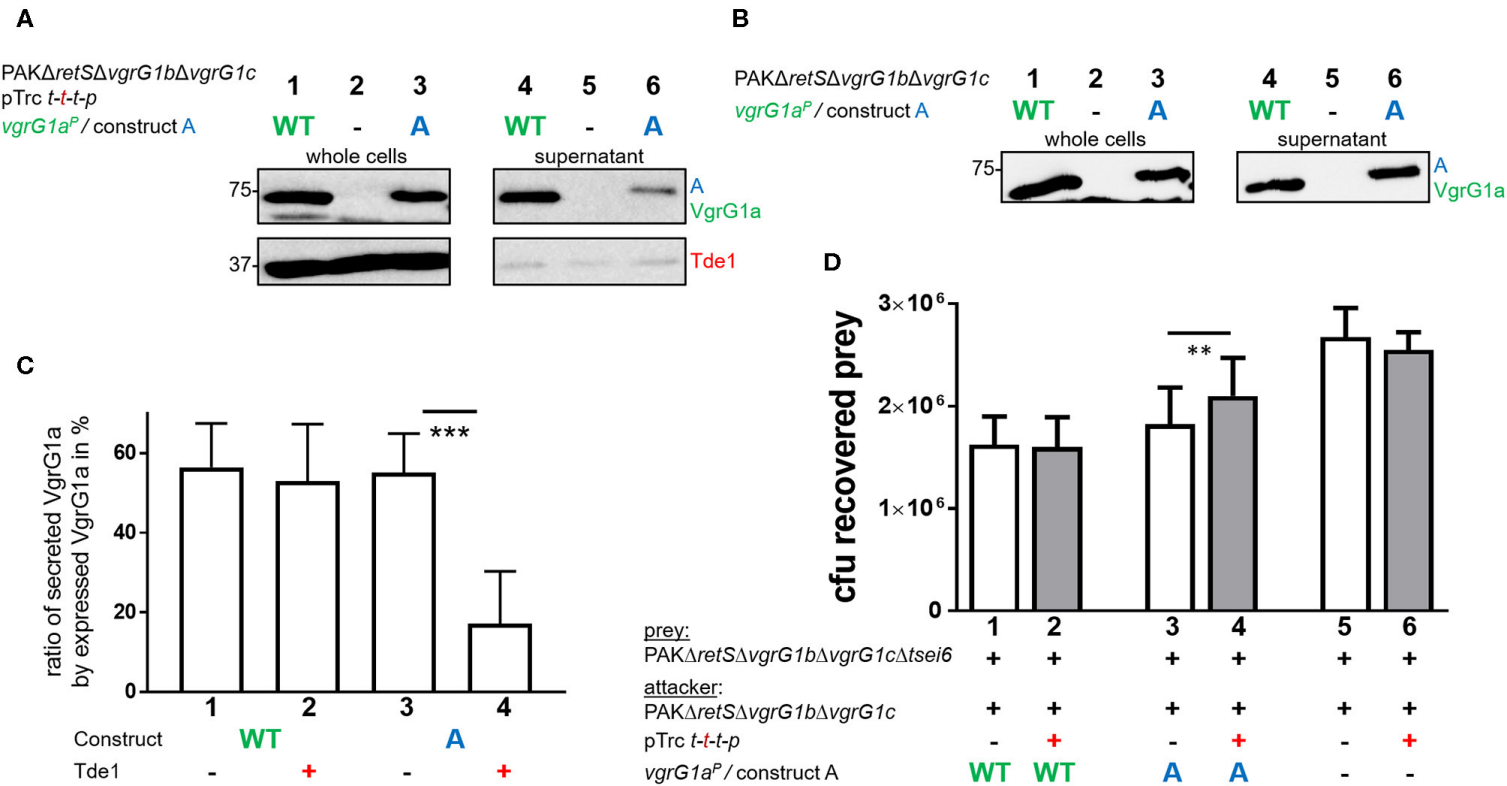
Presence of Tde1 interferes with delivery of construct A and the VgrG1a-dependent effector Tse6. **(A,B)** Sections of western blots from secretion assays of PAKΔ*retS*Δ*vgrG1b*Δ*vgrG1c* expressing native VgrG1a^*P*^ (WT) or chimera A **(A)**. In A, *tap1-tde1-tdi1-paar* is co-expressed from pTrc200, but not in **(B)**. Antibodies were used against VgrG1abc and Tde1 as indicated on the right. **(C)** Percentage of secreted VgrG1a^*P*^ (WT) or construct A **(A)** in absence (–) or presence (+) of *tap1-tde1-tdi1-paar*. Intensity of bands corresponding to VgrG1a^*P*^ was measured using ImageJ and plotted. Dunnett's multiple comparisons test was conducted with ***p < 0.001. **(D)** Plot of recovered cfu of prey strain PAKΔ*retS*Δ*vgrG1b*Δ*vgrG1c*Δ*tsei6::lacZ* after contact with attacker strain that expressed VgrG1a^*P*^ (WT) or construct A **(A)** in absence (–) or presence (+) of *tap1-tde1-tdi1-paar* (*t-t-t-p*) from pTrc200 from five independent experiments. Spots were incubated for 5 h at 37°C in a 5:1 ratio. One-Way ANOVA analysis with Sidak's multiple comparisons test was conducted between the datasets obtained from the two genetically same strain backgrounds with **p < 0.01.

Despite our data suggesting that Tde1 may interact with and stabilize chimeric VgrG1a spikes in *P. aeruginosa*, and that at least one chimera (construct A) is secreted, we did not find conditions which resulted in effective Tde1 secretion. There are a few observations that may explain this controversy. First, we noticed an impact on the secretion efficiency of construct A in presence of Tap1-Tde1-Tdi1-PAAR (Figures 5A,B, compare lanes 6). We quantified these data by measuring the intensities of the corresponding bands from the supernatant fractions, and from three independent experiments, using ImageJ (Figure 5C). Approximately 60% of the produced VgrG1a^*P*^ and construct A are secreted into the supernatant in absence of Tap1-Tde1-Tdi1-PAAR (lanes 1 and 3). However, whereas in the presence of Tap1-Tde1-Tdi1-PAAR, VgrG1a^*P*^ secretion level is unaffected (lane 2), the secretion of construct A drops to 20% (lane 4). This is a remarkable finding which suggests that Tap1-Tde1-Tdi1-PAAR does interfere with construct A secretion but not with VgrG1a^*P*^ and this correlates with the evidence that Tap1-Tde1-Tdi1-PAAR interacts with construct A but not with VgrG1a^*P*^.

Since construct A secretion is impacted by the presence of Tap1-Tde1-Tdi1-PAAR, we hypothesized that delivery of the VgrG1a^*P*^-dependent PAAR effector Tse6 into prey cells would be affected as well. In WT cells, VgrG1a^*P*^ is able to drive killing of Tse6-sensitive cells as shown previously (Hachani et al., 2014). Since construct A secretion is similar to that of VgrG1a^*P*^, one would expect a similar killing of Tse6-sensitive cells, except if the presence of Tap1-Tde1-Tdi1-PAAR interferes with construct A to bind and deliver Tse6. In a competition assay, we used a prey strain lacking *tsei6* which has no longer immunity to Tse6, while the attacker uses either VgrG1a^*P*^ or construct A in presence or absence of Tap1-Tde1-Tdi1-PAAR (Figure 5D). Note that a catalytic mutant of Tde1 was also used to rule out any toxic effect on the prey in case Tde1 was delivered even in small amounts.

The number of recovered prey cells (cfu) was lower when incubated with strains expressing VgrG1a^*P*^ (Figure 5D, lane 1) than with strains lacking VgrG1a^*P*^ (lane 5), confirming that Tse6 delivery depends on VgrG1a^*P*^ (Hachani et al., 2014). Neither of these outcomes was affected by Tde1 presence (lanes 2 and 6). When the attacking strains express construct A but no Tde1 (lane 3), Tse6-dependent killing is reflected by low prey survival similar to WT levels. This confirms that not only is construct A secreted, but it also drives Tse6 delivery into prey cells, corroborating its functionality despite the additional C-terminal residues from VgrG1^*A*^. Remarkably, co-expression of Tap1-Tde1-Tdi1-PAAR (lane 4) leads to an intermediate phenotype suggesting that Tse6 delivery is affected but not completely abrogated. This correlates with the fact that construct A is still secreted but in smaller amounts (Figure 5A, lane 6), which would mean that secretion of the whole VgrG-PAAR complex is affected. Further, this suggests that either binding of Tap1-Tde1 to the tip composed of construct A does not entirely prevent Tse6 secretion, or that only a fraction of construct A is bound by Tap1-Tde1 and others which are associated with Tse6 could deliver Tse6.

## Discussion

Bacterial protein secretion systems are highly specific and proteins secreted by a given secretion type (T1SS-T7SS) usually fail to be recognized by another type due to the lack of appropriate secretion motifs (Voulhoux et al., 2000; Filloux, 2010, 2011). Even proteins secreted by the same type but by distinct systems within the same species would usually fail to be effectively released (Ball et al., 2002). Despite such strong selectivity several studies have successfully managed to reprogram secretion by engineering chimeric effectors or secretion machines (Nicolay et al., 2015; van Ulsen et al., 2018). The present study aimed at evaluating potential venues to redirect a heterologous cargo effector from *A. tumefaciens* to the T6SS of *P. aeruginosa*. We reasoned that a T6SS spike/VgrG could accommodate to the endogenous T6SS machine through its conserved domain, while the additional variable domain could be manipulated to adapt recognition of a heterologous effector. With this in mind we hypothesized that specific interactions between the effector Tde1 and the appropriate C terminal region of its cognate VgrG1^*A*^ might be sufficient to attach the effector to a chimeric but heterologous VgrG spike. We constructed three chimeras made of the *P. aeruginosa* VgrG1a^*P*^ and the Tde1 binding amino acids from VgrG1^*A*^, but none of them was able to complete Tde1 delivery in *P. aeruginosa*.

**Figure 6:**
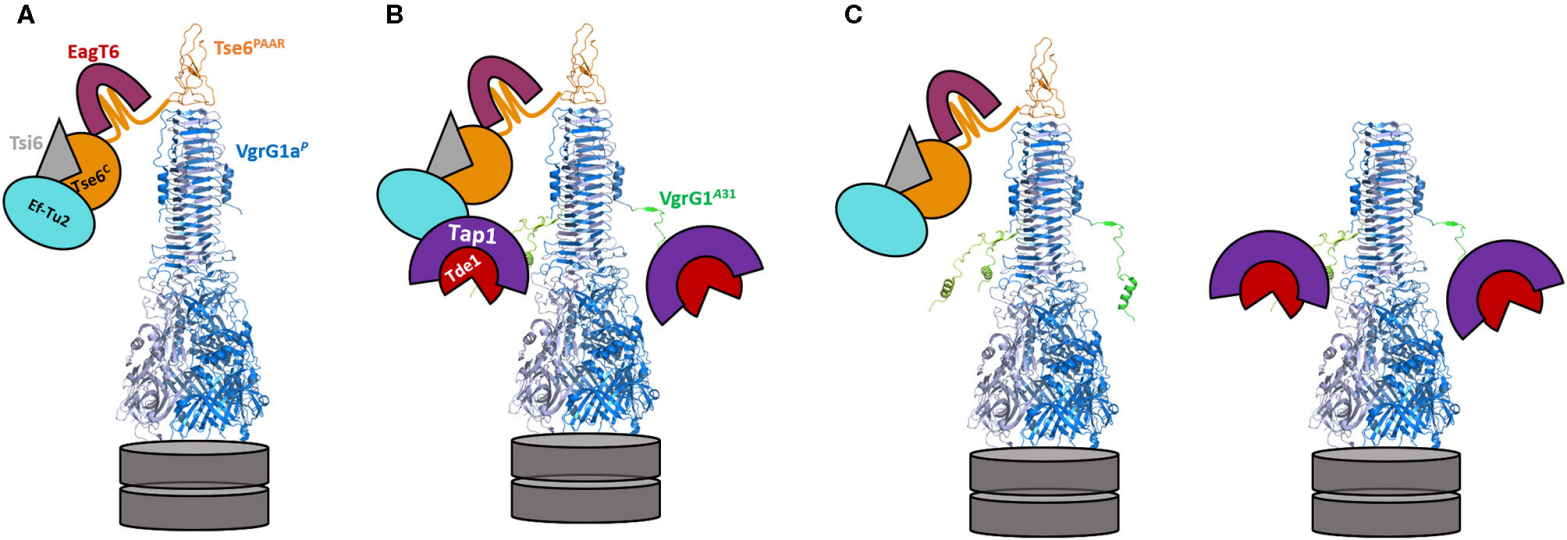
Model of the decoration of the VgrG1a/chimera A-tip in absence and presence of Tap-Tde1. VgrG1a^*P*^ is represented as a blue trimer (pdb: 4mtk), topped by the PAAR domain of Tse6 (orange) with the C-terminal domain of Tse6 floating around the tip bound to its cognate immunity Tsi6 (gray). The chaperone EagT6 (magenta) and the elongation factor EfTu2 (cyan) additionally bind the C-terminal toxin domain of Tse6. **(A)** Configuration of the VgrG1a tip in the parental strain. **(B)** In cells expressing construct A, VgrG1a is extended by 31 aa originally from VgrG1^*A*^ (green) resulting in an extended VgrG spike protein. In the presence of Tap (purple) and Tde1 (red), three Tap1-Tde1-complexes bind to each of the C-terminal extensions of chimera A (only two are shown here) leading to a sterically crowded complex tip. **(C)** Two construct A spikes coexist in *P. aeruginosa* cells. One complex is comparable to the WT spike with Tse6 binding to the three last β-strands of construct A, resulting in a secretion-competent T6SS tip. A second complex interacts with the Tap1-Tde1-complex but not with Tse6 and this complex cannot be secreted resulting in less overall secretion of construct A in comparison to VgrG1a spikes.

Although highly complex in terms of interpretation, several findings from our study could be explained with a concept based on steric hindrance as sketched in Figure 6. In an original *P. aeruginosa* context (Figure 6A), VgrG1a binds and delivers its cognate Tse6 PAAR effector, while EagT6 (magenta) and the elongation factor 2 (Ef2, cyan) also tightly interact with and sterically occupy the space around the VgrG tip (Whitney et al., 2015; Quentin et al., 2018). However, when chimera A is expressed, the only chimera which *per se* is secreted by *P. aeruginosa*, the same Tse6-associated components are interacting with the tip, since none of the interaction surfaces between VgrG1a and the Tse6 PAAR domain was modified. Additionally, three Tap1-Tde1-complexes (purple-red and only two of them depicted in the figure) could also bind to each monomer of the chimera A spike complex, with such interaction having been confirmed by the bacterial-two-hybrid assay (Figure 4A). In all, we suggest that the steric hindrance due to a wealth of binding partners around the VgrG tip might be a limitation factor (Figure 6B). Since we observed a hampered construct A secretion (Figure 5A, lane 6) and Tse6 delivery (Figure 5D, lane 4) in the presence of Tap1-Tde1-complexes, one might also suggest that two complexes coexist in *P. aeruginosa* cells (Figure 6C). A set of construct A proteins might form a functional T6SS spike capped by Tse6 resulting in secretion of the complex. A second set of construct A might be bound to the Tap1-Tde1-complex, which might form a VgrG trimer, but does not bind Tse6, thus not leading to a functional trimer that is being secreted. One might suggest the production of both WT VgrG1a and chimera A within one background to facilitate the production of a heterotrimer that could secrete both Tse6 as a PAAR and Tde1 as a cargo effector. However, production of such heterotrimer would be challenging as it would consist of three VgrG proteins with an unknown ratio of WT VgrG1a and chimera A. Furthermore, in a previous study we saw that in presence of two VgrG1a species, WT VgrG1a and modified VgrG1a (Wettstadt and Filloux, 2020), WT VgrG1a was secreted in higher amounts than the modified VgrG1a suggesting a preferred formation of WT VgrG1a spikes to be secreted.

Our results may emphasize a major limitation of T6SS-mediated effector delivery, which is the available space around the VgrG tip and probably within the T6SS membrane complex. Recent studies visualized the baseplate structure (Nazarov et al., 2018) and the membrane complex (Durand et al., 2015) of the T6SS and cavities around the spike complexes which were unoccupied and proposed to allow the accommodation of effector proteins. However, it is not clear whether there is enough space to accommodate additional and folded cargo and a wealth of decorations around the VgrG tip would prevent the fit into the membrane complex. It is also to take into consideration that the dimension from the T6SS machine originating from *V. cholerae* and *E. coli* might not be similar for the H1-T6SS from *P. aeruginosa*.

Our study highlights the fine balance between the functionality of the spike, e.g., its ability to perforate the cell envelope once embedded in the T6SS membrane complex and thus its delivery into the extracellular medium, and its capacity to bind a cargo effector and drive its secretion. Previous studies in *Serratia marcescens, Acinetobacter baylyi*, and *A. tumefaciens* demonstrated that at least one VgrG protein and its cognate PAAR need to assemble to form a functional T6SS tip (Shneider et al., 2013; Bondage et al., 2016; Cianfanelli et al., 2016; Wu et al., 2020). In the case of *S. marcescens*, three such assemblies can form: VgrG1 and its cognate PAAR; VgrG2 and Rhs1 or VgrG2 and Rhs2. Each of the two PAAR domains of the Rhs effectors can top VgrG2 and thus form a functional spike complex. Similarly, in *A. tumefaciens* two such assemblies were confirmed: VgrG1 with PAAR delivering Tde1 and VgrG2 with the PAAR effector Tde2 (Bondage et al., 2016). In *P. aeruginosa*, it was previously demonstrated, that each VgrG associated with the H1-T6SS, VgrG1a, VgrG1b, or VgrG1c, can associate with a cognate PAAR effector, Tse6, Tse7, and Tse5, respectively (Hachani et al., 2014). Here, we showed in *P. aeruginosa* that presence of the full length VgrG1a is required to form a functional H1-T6SS spike while any chimera lacking parts of the C-terminal residues of VgrG1a (construct B or C) was not able to perform the same function. This might suggest that the C-terminal residues of VgrG1a are required for specific interactions with its cognate PAAR effector Tse6, and ultimately the presence of the PAAR in the spike would be needed for effective secretion of the whole spike. This explanation is consistent with recent findings that effector loading onto its cognate VgrG spike activates T6SS assembly (Liang et al., 2019; Wu et al., 2020). This is also supported by data showing that the three last β-sheets of VgrG1b specifically interact with the cognate PAAR effector Tse7 (Shneider et al., 2013; Pissaridou et al., 2018). Yet, the cargo effectors from *P. aeruginosa* and *E. coli*, PldA, PldB and Tle1, that do not contain N-terminal PAAR domains, were shown to specifically bind to the C-terminal domains of their cognate VgrGs, VgrG4b, VgrG5, and VgrG1, respectively (Flaugnatti et al., 2015; Wettstadt et al., 2019). In this case it is likely that the T6SS spike would be completed by a standalone PAAR domain (Wood et al., 2019a).

Our study confirmed that heterologous secretion cannot easily result from simple and straightforward genetic manipulations and the recognition of a secreted effector by its own machinery has likely resulted from a longstanding co-evolution which guarantees specificity. This way it would be hard to hijack the process and only intended effectors are released upon specific conditions, avoiding also any leakage of intracellular proteins. In the T6SS where C terminal motifs in the VgrG spike are proven to confer effector recognition specificity, search for motifs within the effector *per se* has not given what one could consider a universal T6SS motif. Yet, a conserved motif has been found in a subset of T6SS effectors, which has been called the MIX motif (Marker for type sIX effectors) (Salomon et al., 2014). In several other T6SS effectors, a different but conserved domain is also found at the N terminus, that has been called FIX (Jana et al., 2019). Whether these domains could be used to engineer chimeric T6SS effectors that will be retargeted to heterologous T6SS machine is yet to be fully investigated.

## Data Availability Statement

All datasets generated for this study are included in the article/Supplementary Material.

## Author Contributions

SW contributed to conceptualization, investigation, methodology, and writing the original draft. E-ML and AF were responsible for the conceptualization, funding acquisition, supervision, and reviewing and editing the manuscript. All authors contributed to the article and approved the submitted version.

## Conflict of Interest

The authors declare that the research was conducted in the absence of any commercial or financial relationships that could be construed as a potential conflict of interest.
