# Supplementary material for: Solving the Puzzle: Connecting a Heterologous Agrobacterium tumefaciens T6SS Effector to a Pseudomonas aeruginosa Spike Complex
Source: Front Cell Infect Microbiol. 2020 Jun 23;10:291. doi: 10.3389/fcimb.2020.00291 (PMC7324665; doi:10.3389/fcimb.2020.00291)
Supplement: Supplementary file 1 [file Table_1.DOCX]

Table S1

Bacterial strains used in this study.

| Strain | Characteristics | Source |
| --- | --- | --- |
| P. aeruginosa |  |  |
| PAKΔretS | Wild type *P. aeruginosa* PAK strain with a deletion in *retS* (PA4856) | Lab collection |
| PAKΔretSΔtse6tsi6 | PAKΔ*retS* with a deletion in *tse6* (PA0093) and *tsi6* (PA0092) | This study |
| PAKΔretSΔtse6tsi6 ::lacZ | PAKΔ*retS* with a deletion in *tse6-tsi6* (PA0093- PA0092), chromosomal insertion of *lacZ* at *att* site | This study |
| PAKΔretSΔvgrG1a | PAKΔ*retS* with a deletion in *vgrG1a* (PA0091) | (Hachani et al. 2011) |
| PAKΔretSΔvgrG1a ΔvgrG1bΔvgrG1c | PAKΔ*retS* with deletions in *vgrG1a* (PA0091), *vgrG1b* (PA0095) and *vgrG1c* (PA2685) | (Hachani et al. 2011) |
| PAKΔretS::vgrG1a-vgrG1^A31^ | PAKΔ*retS* with an insertion of gene portion corresponding *vgrG1^A31^* before *vgrG1a* STOP codon | This study |
| PAKΔretS::vgrG1a^605^-vgrG1^A31^ | PAKΔ*retS* with a substitution of gene portion corresponding to *vgrG1a^P39^* with gene portion for *vgrG1^A31^* | This study |
| PAKΔretS::vgrG1a^614^-vgrG1^A21^ | PAKΔ*retS* with a substitution of gene portion corresponding to *vgrG1a^P28^* with gene portion for *vgrG1^A21^* | This study |
| PAKΔretSΔvgrG1bΔvgrG1c | PAKΔ*retS* with deletions in *vgrG1b* (PA0095) and *vgrG1c* (PA2685) | (Hachani et al. 2011) |
| PAKΔretSΔvgrG1b ΔvgrG1cΔtse6tsi6 | PAKΔ*retS* with deletions in *vgrG1b* (PA0095), *vgrG1c* (PA2685), *tse6-tsi6* (PA0093- PA0092) | This study |
| PAKΔretSΔvgrG1b ΔvgrG1cΔtse6tsi6 ::lacZ | PAKΔ*retS* with deletions in *vgrG1a* (PA0091), *vgrG1c* (PA2685), *tse6-tsi6* (PA0093- PA0092), chromosomal insertion of *lacZ* at *att* site | This study |
| PAKΔretSΔvgrG1b ΔvgrG1c::vgrG1a-vgrG1^A31^ | PAKΔ*retS* with deletions in *vgrG1b* (PA0095) and *vgrG1c* (PA2685), with an insertion of gene portion corresponding *vgrG1^A31^* before *vgrG1a* STOP codon | This study |
| PAKΔretSΔvgrG1b ΔvgrG1c::vgrG1a^605^-vgrG1^A31^ | PAKΔ*retS* with deletions in *vgrG1b* (PA0095) and *vgrG1c* (PA2685), with a substitution of gene portion corresponding to *vgrG1a^P39^* with gene portion for *vgrG1^A31^* | This study |
| PAKΔretSΔvgrG1b ΔvgrG1c::vgrG1a^614^-vgrG1^A21^ | PAKΔ*retS* with deletions in *vgrG1b* (PA0095) and *vgrG1c* (PA2685), with a substitution of gene portion corresponding to *vgrG1a^P28^* with gene portion for *vgrG1^A21^* | This study |
| A. tumefaciens |  |  |
| A. tumefaciens C58 | C58 Wild type strain | Gift from Eugene Nester |
| E. coli |  |  |
| DH5α | F– *endA1 glnV44 thi-1 recA1 relA1 gyrA96 deoR nupG purB20 φ80dlacZΔM15 Δ(lacZYA-argF)U169, hsdR17(rK–mK+), λ–* | ThermoFisher |
| Sm10λpir | Host strain for Mini-CTX1 replication: thi thr leu tonA lacY supE *recA*::RP4-2-Tc::Mu (Km^R^) λpir | (Miller, Mekalanos 1988) |
| CC118λpir | Host strain for pKNG101 replication: Δ(ara-leu) *araD* ΔlacX74 *galE galK-phoA20 thi-1 rpsE rpoB argE* (Sm^R^) *recA1* Rfr λpir | (Herrero et al. 1990) |
| DHM1 | *cya-854 recA1 gyrA96 (NaIR) thi1 hsdR17 spoT1 rfbD1 glnV44*(AS) | (Karimova et al. 1998) |
| 1047 | Helper strain for conjugation: (Km^R^), *oriColE1 RK2- Mob+ RK2-Tra+* | (Figurski, Helinski 1979) |
