# Supplementary material for: Solving the Puzzle: Connecting a Heterologous Agrobacterium tumefaciens T6SS Effector to a Pseudomonas aeruginosa Spike Complex
Source: Front Cell Infect Microbiol. 2020 Jun 23;10:291. doi: 10.3389/fcimb.2020.00291 (PMC7324665; doi:10.3389/fcimb.2020.00291)
Supplement: Supplementary file 2 [file Table_2.DOCX]

**Table S2**

**Plasmids used in this study**.

| Plasmid | Characteristics | Source |  |
| --- | --- | --- | --- |
| Vectors for chromosomal mutagenesis | | | |
| pRK2013 | Self-transmissible helper plasmid for three-partner conjugations, KmR | (Figurski, Helinski 1979) |  |
| pKNG101 | Non-replicative suicide vector to alter *P. aeruginosa* chromosome. *ori6K, mobRK2, sacB*, SmR | Lab collection |  |
| pKNG101::vgrG1a-vgrG1^A31^ | pKNG101 suicide plasmid to integrate 93 bp of *vgrG1^A^* before STOP codon of *vgrG1a^P^*, SmR | This study |  |
| pKNG101::vgrG1a^605^-vgrG1^A31^ | pKNG101 suicide plasmid to substitute final 114 bp of *vgrG1a^P^* with 93 bp from *vgrG1^A^*, SmR | This study |  |
| pKNG101::vgrG1a^614^-vgrG1^A31^ | pKNG101 suicide plasmid to substitute final 95 bp of *vgrG1a^P^* with 66 bp from *vgrG1^A^*, SmR | This study |  |
| pKNG101Δtse6tsi6 | pKNG101 suicide plasmid to *tse6-tsi6*, an effector-immunity module, SmR | This study |  |
| pKNG101ΔtssB1 | pKNG101 suicide plasmid to delete *tssB1*, an essential sheath component of the H1-T6SS, SmR | (Lossi et al. 2012) |  |
| Cloning vectors | | | |
| pCR®-Blunt II-TOPO® | Subcloning vector for blunt-ended inserts, KmR | ThermoFisher |  |
| Bacterial-two-hybrid vectors | | | |
| pKT25 | BTH vector for C-terminal fusions with the T25 fragment of *Bordetella pertussis* adenylyl cyclase (CyaA), KmR | (Karimova et al. 1998) |  |
| pUT18C | BTH vector for C-terminal fusions with the T18 fragment of *B. pertussis* CyaA, ApR | (Karimova et al. 1998) |  |
| pKT25-zip | BTH vector producing a C-terminal leucine zipper fusion with the T25 fragment of *B. pertussis* CyaA, KmR | (Karimova et al. 1998) |  |
| pUT18C-zip | BTH vector producing a C-terminal leucine zipper fusion with the T18 fragment of *B. pertussis* CyaA, ApR | (Karimova et al. 1998) |  |
| pKT25 vgrG1a | BTH vector producing a C-terminal fusion of VgrG1a to T25-fragment of *B. pertussis* CyaA, KmR | Lab collection |  |
| pUT18C vgrG1a | BTH vector producing a C-terminal fusion of VgrG1a^P^ to T18-fragment of *B. pertussis* CyaA, AmR | Lab collection |  |
| pKT25 vgrG1a-vgrG1^A31^ | BTH vector producing a C-terminal fusion of VgrG1a-vgrG1^A31^ to T25-fragment of *B. pertussis* CyaA, KmR | This study |  |
| pUT18C vgrG1a-vgrG1^A31^ | BTH vector producing a C-terminal fusion of VgrG1a-vgrG1^A31^ to T18-fragment of *B. pertussis* CyaA, AmR | This study |  |
| pKT25 vgrG1a^605^-vgrG1^A31^ | BTH vector producing a C-terminal fusion of VgrG1a^605^-vgrG1^A31^ to T25-fragment of *B. pertussis* CyaA, KmR | This study |  |
| pUT18C vgrG1a^60^5-vgrG1^A31^ | BTH vector producing a C-terminal fusion of VgrG1a^605^-vgrG1^A31^ to T18-fragment of *B. pertussis* CyaA, AmR | This study |  |
| pKT25 vgrG1a^614^-vgrG1^A31^ | BTH vector producing a C-terminal fusion of VgrG1a^614^-vgrG1^A31^ to T25-fragment of *B. pertussis* CyaA, KmR | This study |  |
| pUT18C vgrG1a^614^-vgrG1^A31^ | BTH vector producing a C-terminal fusion of VgrG1a^614^-vgrG1^A31^ to T18-fragment of *B. pertussis* CyaA, AmR | This study |  |
| pKT25 vgrG1^A^ | BTH vector producing a C-terminal fusion of VgrG1^A^ to T25-fragment of *B. pertussis* CyaA, KmR | This study |  |
| pUT18C vgrG1^A^ | BTH vector producing a C-terminal fusion of VgrG1a^A^ to T18-fragment of *B. pertussis* CyaA, AmR | This study |  |
| pKT25 tap1-tde1(HADA) | BTH vector producing Tap1 and a C-terminal fusion of Tde1 to T25-fragment of *B. pertussis* CyaA, KmR | This study |  |
| pUT18C tap1-tde1(HADA) | BTH vector producing Tap1 and a C-terminal fusion of Tde1 to T18-fragment of *B. pertussis* CyaA, AmR | This study |  |
| pKT25 tde1(HADA) | BTH vector producing and a C-terminal fusion of Tde1 to T25-fragment of *B. pertussis* CyaA, KmR | This study |  |
| pUT18C tde1(HADA) | BTH vector producing and a C-terminal fusion of Tde1 to T18-fragment of *B. pertussis* CyaA, AmR | This study |  |
| Expression vectors | | | |
| pTrc200 | Broad host range pVS1 derivative plasmid, lacIq, pTrc promoter, SmR/SpR | (Schmidt-Eisenlohr et al. 1999) |  |
| p tap1-tde1-tdi1-paar | pTrc200 producing Tap1-Tde1-Tdi1-PAAR from *A. tumefaciens*, SmR/SpR | (Ma et al. 2014) |  |
| p tap1-tde1(HADA)-tdi1-paar | pTrc200 producing Tap1-Tde1(HADA)-Tdi1-PAAR from *A. tumefaciens*, SmR/SpR | (Ma et al. 2014) |  |
| pRL662 | Broad host range pBBR1-derivative vector with a constitutive PLAC promoter, GmR | (Ma et al. 2014) |  |
| p vgrG1^A^ | pRL662 producing full-length VgrG1^A^, GmR | (Ma et al. 2014) |  |
| miniCTX::lacZ | Mini-CTX1 harbouring the *lacZ* with a constitutive promoter, TcR | (Becher, Schweizer 2000) |  |
