# Supplementary material for: Solving the Puzzle: Connecting a Heterologous Agrobacterium tumefaciens T6SS Effector to a Pseudomonas aeruginosa Spike Complex
Source: Front Cell Infect Microbiol. 2020 Jun 23;10:291. doi: 10.3389/fcimb.2020.00291 (PMC7324665; doi:10.3389/fcimb.2020.00291)
Supplement: Supplementary file 3 [file Table_3.DOCX]

**Table S3. Primers used in this study**

| Construct | Primer name | Code | Sequence |
| --- | --- | --- | --- |
| Vector-screening primers | | | |
| pKNG101 | pKNG101_F | OAL682 | CCCTGGATTTCACTGATGAG |
|  | pKNG101_R | OAL683 | CATATCACAACGTGCGTGGA |
| pTrc200 | pTrc200_F | OAL2848 | tttgcgccgacatcataacg |
|  | pTrc200_R | OAL2849 | gccaggcaaattctgttttatca |
| pUT18C | pUT18C_F | OAL190 | GAAGTTCTCGCCGGATGTACTGGAAACGGT |
|  | pUT18C_R | OAL191 | TTGGCGGGTGTCGGGGCTGGCTTAACTATG |
| pKT25 | pKT25_F | OAL192 | TGGCGAAAGGGGGATGTGCTGCAAGGCGAT |
|  | pKT25_R | OAL193 | GTGACCAGCGGCGATTCGGTGACCGATTAC |
| Mutagenesis primers | | | |
| pKNG101::vgrG1a-shuttles | *vgrG1a*_F | OAL3735 | CGAGGAGATCTGGACCGAC |
|  | *vgrG1a*_R | OAL3736 | AAGGACATCGATCCTGCCG |
| pKNG101::vgrG1a-vgrG1^A31^ | *vgrG1a-vgrG1^A31^­*_upF | OAL3125 | CGGCGAAGGGCAACCAGATCAACTTC |
|  | *vgrG1a-vgrG1^A31^­*_upR | OAL3124 | GAAGTTGATCTGGTTGCCCTTCGCC |
|  | *vgrG1a-vgrG1^A31^­*_dnF | OAL3119 | CATCGACACCGGCAACCAGATCAA |
|  | *vgrG1a-vgrG1^A31^­*_dnR | OAL3118 | TTGATCTGGTTGCCGGTGTCGAT |
| pKNG101::vgrG1a^605^-vgrG1^A31^ | *vgrG1a^605^-vgrG1^A31^­*_upF | OAL3121 | TCAGGATTTGATGAGGCGGC |
|  | *vgrG1a^605^-vgrG1^A31^­*_upR | OAL3120 | CGCCGCCTCATCAAATCCTGAC |
| pKNG101::vgrG1a^614^-vgrG1^A31^ | *vgrG1a^614^-vgrG1^A21^­*_upF | OAL3699 | CCGGCGGCCCAGGTGAAAG |
|  | *vgrG1a^614^-vgrG1^A21^­*_upR | OAL3698 | CTGGGCCGCCGGAATTGAG |
| pKNG101 Δtse6tsi6 | Δ*tse6tsi6*_upF | OAL3348 | TGAGGCGGCGGCATCCTGGCT |
|  | Δ*tse6tsi6*_upR | OAL3349 | AGCTGACCCGAGTACCACTGCAC |
|  | Δ*tse6tsi6*_dnF | OAL3350 | CGGTGCAGTGGTACTCGGGTC |
|  | Δ*tse6tsi6*_dnR | OAL3351 | ACATTCCGTAAAGCTTTTGTCCAG |
|  | Δ*tse6tsi6*_exF | OAL3352 | AGTTATACATCCACGCCGAGC |
|  | Δ*tse6tsi6*_exR | OAL3353 | GAAAGGGGAGATGCGTGACA |
| Bacterial-two-hybrid primers | | | |
| vgrG1a^P^ | *vgrG1a^P^*_F | OAL3973 | CCGGTCTAGAGCAACTGACCCGCCTGGTC |
|  | *vgrG1a^P^*_R | OAL1741 | GCGCGGAATTCTCAGCCCTTCGCCGGCGG |
| vgrG1^A^ | *vgrG1^A^*_F | OAL3975 | CCGGTCTAGAGAACGACCAGCCTTCGGTT |
|  | *vgrG1^A^*_R | OAL3974 | ATATGGATCCTCAAATCCTGACAAACGGTG |
| tap1-tde1(HADA) | *tap1-tde1*_F | OAL3722 | gcgcTCTAGAATGAACGACACACCCATAATTGATC |
|  | *tap1-tde1*_R | OAL3723 | ccggggatccAAAGACACCGGGACGTCAGG |
| tde1(HADA) | *tde1*_F | OAL3743 | gcgcTCTAGAATGAGTGCGACGACAACTG |
